# Multi-Target Profiling of Antioxidant Compounds, Including Repurposing and Combination Strategies

**DOI:** 10.3390/antiox14020220

**Published:** 2025-02-15

**Authors:** Roberta Rocca

**Affiliations:** Department of Health Sciences, University Magna Græcia, 88100 Catanzaro, Italy; rocca@unicz.it

## 1. Introduction

Multifactorial diseases, such as cancer, neurodegenerative disorders, and stroke, present significant challenges in modern medicine due to their complex origins and the absence of definitive treatments [1]. These diseases result from a combination of genetic, environmental, and lifestyle factors, making them particularly complicated to treat with single-target drugs [2]. Among the multiple pathological mechanisms involved, oxidative stress has emerged as a key factor in their progression. The excessive production of reactive oxygen species (ROSs) and the resulting cellular damage contribute to inflammation, DNA mutations, and neuronal degeneration, further exacerbating disease symptoms [3].

Given the limited effectiveness of current treatments, researchers have increasingly embraced multi-targeting drug approaches as a promising therapeutic strategy [4]. Unlike traditional single-target drugs, which may only provide symptomatic relief or limited benefits, multi-targeting agents have the potential to interact with multiple disease pathways simultaneously [2]. By targeting oxidative stress, inflammation, and other key contributors, these compounds could provide a more integrated and potent approach to disease management. It is also true that compounds targeting oxidative stress may have additional, unexplored mechanisms of action, making them potential candidates for inclusion in multi-target strategies.

In this context, in silico techniques have emerged as powerful tools in rational drug design within the field of medicinal chemistry. Computational methods such as molecular docking, virtual screening, and pharmacophore modeling allow researchers to predict how new compounds will interact with multiple biological targets. These techniques not only expedite the drug discovery process, but also help reduce costs and enhance the chances of identifying promising therapeutic candidates before advancing to experimental validation [5].

A central focus in the development of multi-target drugs can be considered the design and synthesis of molecules that combine both antioxidant and anti-inflammatory properties. By reducing oxidative damage and regulating inflammatory pathways, these compounds show great potential for treating a wide range of multifactorial diseases [6]. Recent advancements in medicinal chemistry have led to the creation of innovative molecular frameworks that integrate these beneficial properties, opening the door to groundbreaking therapeutic solutions.

This Special Issue explores the role of oxidative stress in multifactorial diseases, highlights the potential of multi-targeting strategies, and discusses how in silico drug design is revolutionizing the search for effective treatments. By combining computational approaches with experimental validation, researchers aim to develop next-generation therapeutics that can improve patient outcomes and address the urgent need for innovative treatments in complex diseases.

## 2. Overview of Published Articles

Chemoprevention is a vital aspect of modern health management, and Kim et al. [Contribution 1] underscore the multitarget potential of *Brassica rapa* doubled haploid (DH) lines enriched with high glucosinolate (GSL) content. Extracts from selected high-GSL DH lines were evaluated for their anticancer activities in human colorectal cancer (CRC) cells, demonstrating anti-proliferative and pro-apoptotic effects. These extracts exerted their effects through the inhibition of multiple signaling pathways, including NF-κB and ERK, leading to reduced nuclear localization of NF-κB p65. Additionally, treatment with the high-GSL DH extracts induced the production of reactive oxygen species in CRC cells. The results suggest that these high-GSL DH lines possess multitarget anticancer properties, making them promising candidates for chemoprevention as a daily vegetable supplement.

Polyphenolic compounds, including flavonoids and non-flavonoids, have demonstrated a wide range of health benefits, including antioxidant, anti-inflammatory, and anticancer properties. Altomare and colleagues [Contribution 2] conducted a comprehensive review of the literature data, analyzing clinical and preclinical studies from the past decade. Their study highlights the potential of polyphenols as adjunctive agents in chemotherapy, particularly in mitigating oxidative stress-related toxicity induced by anticancer drugs. While pharmacological data support their role in inhibiting cancer progression, clinical studies have yielded mixed results, with some polyphenols failing to demonstrate protective effects in human trials. A major challenge lies in their metabolic stability and bioavailability, which could be enhanced through advanced delivery systems, such as nanotechnology-based formulations. Further research is essential to establish optimal dosing and delivery strategies, maximizing the therapeutic potential of polyphenols in cancer treatment.

An extensive review of recent advancements in the study of antioxidant compounds and their role in bone defect repair was provided by Wang et al. [Contribution 3]. Their findings underscore the potential of site-specific and controlled drug delivery systems to modulate reactive oxygen species (ROS) levels, presenting a promising avenue for enhancing bone regeneration. However, the effectiveness of these strategies is contingent upon addressing key challenges, including targeted delivery, compound stability, and the intricate role of ROSs in the healing process. The authors stress the necessity of continued research to refine delivery mechanisms, ensuring both stability and optimal ROS regulation for improved therapeutic outcomes. Moreover, they highlight the critical need for additional clinical trials to establish the safety and efficacy of integrating antioxidants with biomaterials for potential clinical applications.

Jiang and colleagues [Contribution 4] explored the synergistic effects of combining pregabalin with dexborneol to enhance pain relief while minimizing adverse reactions. Their research demonstrated that dexborneol facilitates pregabalin’s transport to the central nervous system, interrupts chronic pain cycles by reducing HMGB1 and ATP release, and provides antioxidant benefits by modulating lipid metabolism. This dual-drug approach not only strengthened pregabalin’s analgesic effects, but also reduced neuroinflammation and glial cell activation, both of which play critical roles in chronic pain conditions. By acting on multiple biological pathways, dexborneol enabled the use of lower pregabalin doses, thereby decreasing side effects while preserving therapeutic effectiveness. These findings underscore dexborneol’s potential as a valuable adjunct in pain management, presenting a safer and more efficient multitarget therapeutic strategy.

Maeda et al. [Contribution 5] showcased the multitarget potential of apomorphine in steatohepatitis, demonstrating its capacity to suppress ferroptosis, reduce oxidative stress, and stimulate the Nrf2 signaling pathway. Their findings identified ferroptosis as an early event in steatohepatitis progression in PTEN KO mice, emphasizing its importance as a key therapeutic target for preventing disease development. Apomorphine exhibited potent radical trapping activity, surpassing that of known ferroptosis inhibitors, while simultaneously enhancing cellular defense mechanisms through Nrf2-mediated antioxidant responses. This multitarget approach not only reduced hepatocyte cell death, but also alleviated liver inflammation and mitigated fibrosis, indicating its broad-spectrum protective effects. These findings suggest that apomorphine holds promise as a ferroptosis-targeting therapeutic for liver diseases, offering a multifaceted strategy to combat steatohepatitis and its complications.

Sánchez-Alcázar and colleagues [Contribution 6] demonstrated a multitarget therapeutic approach for LIPT1 deficiency by identifying a combination of antioxidants and mitochondrial-boosting agents capable of restoring cellular function. Their study revealed that this cocktail—comprising pantothenate, nicotinamide, vitamin E, thiamine, biotin, and α-lipoic acid—rescued mitochondrial protein lipoylation, improved cell bioenergetics, and reduced iron accumulation and lipid peroxidation. The beneficial effects were attributed to the activation of SIRT3 and mitochondrial unfolded protein response (mtUPR), highlighting a complex interplay between metabolic rescue and mitochondrial homeostasis. This multitarget strategy not only addressed the primary metabolic defects, but also mitigated oxidative stress and cellular damage, underscoring its potential as a therapeutic intervention for patients with LIPT1 mutations. Their findings pave the way for personalized medicine approaches, using patient-derived cell models to optimize treatment efficacy before clinical application.

Sulfodyne^®^, a natural NRF2 agonist, exerts multitarget antiviral and anti-inflammatory effects against SARS-CoV-2 infection. Romeo et al. [Contribution 7] revealed that Sulfodyne^®^ not only activates NRF2, but also suppresses viral replication by regulating ER stress and mTOR signaling, both of which play a crucial role in viral protein synthesis. It also fine-tunes the immune response by reducing excessive type I interferon signaling and preventing hyperinflammation, a key driver of COVID-19 severity. Additionally, Sulfodyne^®^ regulates the epithelial-immune crosstalk, preventing excessive monocyte activation and infiltration, which contribute to lung pathology. These findings highlight Sulfodyne^®^ as a promising therapeutic agent, leveraging multiple mechanisms to combat SARS-CoV-2 pathogenesis and improve disease outcomes.

Artese et al. [Contribution 8] explored a multitarget strategy for cancer treatment by identifying arundinin, a natural compound that simultaneously inhibits HDAC8 and tubulin. By leveraging computational screening and molecular dynamics simulations, they discovered arundinin’s ability to disrupt chromatin organization and tubulin function, both critical to cancer cell survival. The compound effectively induced apoptosis in breast cancer cells by promoting mitochondrial dysfunction and oxidative stress, highlighting its therapeutic potential. Their study underscores the power of dual-target inhibitors in oncology, demonstrating how a single molecule can modulate multiple pathways essential for cancer progression. This research reinforces the role of natural compounds in drug discovery and opens new avenues for innovative, multitarget cancer therapies.

## 3. Conclusions

The studies presented in this Special Issue emphasize the critical role of oxidative stress in multifactorial diseases, and highlight the growing importance of multitarget drug strategies in modern medicine. By addressing multiple pathological mechanisms simultaneously, these approaches offer a more comprehensive and effective means of disease management. Advances in computational drug design have significantly accelerated the identification of promising therapeutic compounds, facilitating the discovery of novel agents with both antioxidant and anti-inflammatory properties. The research showcased here underscores the potential of natural compounds, innovative molecular frameworks, and combination therapies in tackling complex diseases such as cancer, neurodegenerative disorders, and metabolic conditions. Moving forward, continued integration of in silico modeling, experimental validation, and clinical research will be essential in developing next-generation multitarget therapeutics capable of improving patient outcomes.
